# Validation of the portable Bluetooth® Air Next spirometer in patients with different respiratory diseases

**DOI:** 10.1186/s12931-020-01341-z

**Published:** 2020-04-06

**Authors:** Konstantinos P. Exarchos, Athena Gogali, Agni Sioutkou, Christos Chronis, Sofia Peristeri, Konstantinos Kostikas

**Affiliations:** Respiratory Medicine Department, School of Medicine, University of Ioannina, Ioannina, Greece

## Abstract

**Background:**

Chronic respiratory diseases constitute a considerable part in the practice of pulmonologists and primary care physicians; spirometry is integral for the diagnosis and monitoring of these diseases, yet remains underutilized. The Air Next spirometer (NuvoAir, Sweden) is a novel ultra-portable device that performs spirometric measurements connected to a smartphone or tablet via Bluetooth**®**.

**Methods:**

The objective of this study was to assess the accuracy and validity of these measurements by comparing them with the ones obtained with a conventional desktop spirometer. Two hundred subjects were enrolled in the study with various spirometric patterns (50 patients with asthma, 50 with chronic obstructive pulmonary disease and 50 with interstitial lung disease) as well as 50 healthy individuals.

**Results:**

For the key spirometric parameters in the interpretation of spirometry, i.e. FEV_1_, FVC, FEV_1_/FVC and FEF_25–75%_, Pearson correlation and Interclass Correlation Coefficient were greater than 0.94, exhibiting perfect concordance between the two spirometers. Similar results were observed in an exploratory analysis of the subgroups of patients. Using Bland-Altman plots we have shown good reproducibility in the measurements between the two devices, with small mean differences for the evaluated spirometric parameters and the majority of measurements being well within the limits of agreement.

**Conclusions:**

Our results support the use of Air Next as a reliable spirometer for the screening and diagnosis of various spirometric patterns in clinical practice.

## Introduction

Spirometry is a useful tool for diagnosing the cause of unexplained respiratory symptoms and also monitoring patients with known respiratory diseases [1]. It remains the gold standard test for the diagnosis of obstructive airway diseases, including asthma and Chronic Obstructive Pulmonary Disease (COPD). Asthma affects 5–10% of the population [2], while the prevalence of COPD worldwide varies from 7 to 19%, and poses the third leading cause of death [2, 3]. Moreover, for asthma and COPD, spirometry is a valuable aid for assessing disease severity, prognosis and plays a key role in treatment and overall disease management [2–4].

Despite these benefits, spirometry remains largely underused, especially in the offices of primary care physicians [2–5]. This can be attributed to several factors, including bulky and costly spirometric devices, complex interpretation software, the need for frequent calibration of the spirometer, maintenance costs and special training for performing and interpreting spirometry. As a consequence, many primary care physicians refer their patients to hospital settings for spirometric evaluation [6], therefore increasing significantly the cost of these evaluations. During the last few years, several portable spirometers have emerged in the literature, however, only a couple of them have reached the market and appeared in relevant clinical trials [7, 8]. The advent of smart devices (especially smartphones and tablets) has affected the market of portable medical devices, including spirometers. Specifically, the medical device serves as a dedicated electronic device, i.e. a pneumotachograph in the case of a spirometer, and the recorded data are transferred for further processing to the smart device, which is equipped with an accompanying application for further processing. Therefore, the medical device, when coupled with a smartphone, can be stripped from processing power, interface components, size and cost. However, it is of utmost importance that these low-cost portable spirometers are rigorously validated by comparing them with conventional spirometers, using large patient cohorts. It is our view that CE certified medical devices should be tested independently with the results being published in peer-reviewed journals, in order to evaluate the robustness and reproducibility of the devices’ measurements and allow for interpretation of the data by broader audiences, including practicing clinicians and patients (since portable spirometers may also be intended for home use and monitoring).

Another important aspect that should be highlighted, is that once the data are transferred to the smart device, they can be further analyzed using advanced algorithms, e.g. from the field of Artificial Intelligence, in order to perform more complex tasks, e.g. to discriminate between spirometric patterns, identify underlying disease [9, 10]. All the above factors have gradually made spirometry appealing to a wider audience, both from the medical community but also to patients suffering from respiratory diseases. In the current clinical setting a respiratory patient is evaluated with a spirometry at the pulmonologist’s office once every several months, whereas, having a portable spirometer at home allows for frequent “snapshots” of a patient’s respiratory status, where subtle perturbations can be identified earlier and be dealt with. This has been proven particularly useful in a series of chronic conditions such as cystic fibrosis and Amyotrophic Lateral Sclerosis (ALS) [11].

**Fig. 1:**
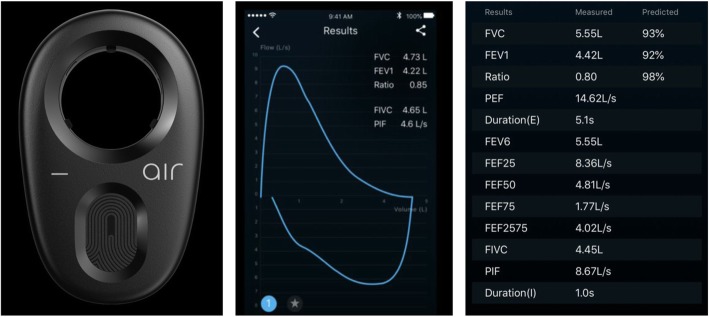
The Air Next spirometer and the reported spirometric parameters

One such portable spirometer that has lately received considerable interest, is the Air Next spirometer by NuvoAir. The Air Next spirometer (NuvoAir, Sweden) is a novel ultra-portable device that performs spirometric measurements connected to a smartphone or tablet via Bluetooth®. Air Next is a certified CE Class IIa Medical Device according to ISO 27782 and 23,747. Through the accompanying application the following indices are stored after a spirometry: forced expiratory volume in 1 s (FEV_1_), forced vital capacity (FVC), FEV_1_/FVC ratio, peak expiratory flow (PEF), duration of spirometry, forced expiratory volume in 6 s (FEV6), mean expiratory flow at 75% (MEF 75), 50% (MEF 50) and 25% (MEF 25) of the vital capacity and forced expiratory flow at 25–75% of the pulmonary volume (FEF 25–75). An appealing characteristic of the Air Next spirometer is that it does not need calibration, due to the fact that the disposable turbines that contain the tachograph come pre-calibrated and have been proven to have only minor deviations for up to approximately 100 uses. Moreover, the flow-volume loop is also presented which is valuable for diagnostic purposes (Fig. 1).

The aim of this study is to validate the portable Air Next spirometer; for this purpose, spirometric data were gathered from a predefined set of patients from the University Hospital of Ioannina. Each patient participating in the study performed spirometry with a conventional spirometer as well as with the Air Next spirometer, and we assessed the agreement between the two spirometers based on certain key spirometric parameters.

## Materials and methods

### Study design

We conducted a descriptive, cross-sectional prospective study at the outpatient clinic of the Respiratory Medicine Department of the University Hospital of Ioannina. We enrolled 200 consecutive patients and healthy volunteers from December 2018 to June 2019, with the following stratification: 50 patients with COPD, 50 patients with asthma, 50 patients with interstitial lung disease and restrictive spirometric pattern and 50 healthy controls. We excluded patients that had any contraindication to perform spirometry: recent hemoptysis of unknown origin, pneumothorax, pulmonary embolism, recent myocardial infarction or unstable angina, aneurysm (cerebral, thoracic, abdominal) or recent eye surgery. Moreover, patients younger than 18 years old or patients that did not provide written informed consent were also excluded from the study.

All patients performed spirometry both with a conventional desktop spirometer currently used by the Respiratory Medicine Department, i.e. the MIR Spirolab (MIR, Italy), and with the study spirometer (Air Next). The desktop spirometer is calibrated according to the manufacturer’s manual, while the Air Next does not need calibration. The order in which the spirometers were used for performing spirometry to each patient in each group was randomized in order to avoid any bias. Measurements with both devices were carried out by trained personnel in a standardized way, according to the ATS/ERS guidelines [12].

A spirometry effort was considered acceptable if the following apply: i) starts from full inhalation, ii) shows minimal hesitation at the beginning of forced expiration, iii) exhibits an explosive start of the forced exhalation, iv) shows no evidence of cough in the first second of forced exhalation and v) meets one of the following criteria that define a valid end-of-test (1 - smooth curvilinear rise of the volume-time tracing to a plateau of at least 1 s’s duration; 2 - if a test fails to exhibit an expiratory plateau, a forced expiratory time of 15 s; or 3 - when the patient cannot or should not continue forced exhalation for valid medical reason) [12]. From each spirometry the following metrics were recorded: FEV_1_ (absolute value in L), FEV_1_% predicted, FVC (absolute value in L), FVC% predicted, FEV_1_/FVC ratio, PEF, MEF_25%_, MEF_50%_, MEF_75%_, FEF_25–75%_.

The study was approved by the Ethics Committee of the University Hospital of Ioannina (meeting 27, topic 10, 05 December 2018). Each participant was informed about the study and provided written informed consent. The consent form was prepared on the basis of the European Union’s template (“GUIDANCE FOR APPLICANTS INFORMED CONSENT - European Commission - Research Directorate-General Directorate L - Science, Economy and Society Unit L3 - Governance and Ethics”), and is in accordance with the requirements of the new General Data Protection Regulation (EU 2016/679).

### Statistical analysis

Descriptive statistics are presented as mean with standard deviation (SD). The agreement and relation between the aforementioned spirometric parameters for both devices were assessed by calculating the Pearson correlation coefficient and the Interclass Correlation Coefficient (ICC). Pearson correlation and ICC were calculated with IBM SPSS statistics, version 24. Moreover, Bland and Altman plots were created to depict the bias between the mean differences for the values obtained by the two spirometric devices with GraphPad Prism v. 8.0.0 (GraphPad Software, San Diego California, USA).

## Results

In this study, 200 patients performed spirometry with a conventional spirometer and with the portable Air Next spirometer. In order to obtain representative results we recruited patients according to the following stratification: 50 patients with asthma, 50 patients with COPD, 50 patients with interstitial lung disease and restrictive spirometric pattern and 50 healthy controls. The following spirometric parameters were recorded for all patients and with both spirometers: FEV_1_, FVC, FEV_1_/FVC, FEF_25–75%_, PEF, MEF_25%_, MEF_50%_ and MEF_75%_. Table 1 contains the key spirometric parameters and their distribution across the four classes with both employed spirometers.

**Table 1:** Key spirometric parameters across the four patient classes, with both spirometers: (1) conventional spirometer and (2) Air Next spirometer

Spirometric parameters					
		**Conventional spirometer**		**Air Next spirometer**	
**Diagnosis**		**FEV**_**1**_**(L)**	**FVC (L)**	**FEV**_**1**_**(L)**	**FVC (L)**
Asthma	Mean	2,15	2,95	2,08	2,85
	Minimum	0,78	1,10	0,82	1,04
	Maximum	4,72	7,06	4,15	5,71
	Std. Deviation	0,82	1,15	0,81	1,06
COPD	Mean	1,96	3,08	1,85	2,91
	Minimum	0,96	1,59	0,90	1,53
	Maximum	3,95	5,53	3,87	4,84
	Std. Deviation	0,63	0,83	0,60	0,72
Normal	Mean	3,07	3,83	3,06	3,73
	Minimum	1,81	2,38	1,73	2,16
	Maximum	4,83	6,18	4,84	6,16
	Std. Deviation	0,69	0,96	0,75	0,99
Restrictive	Mean	2,25	2,90	2,16	2,80
	Minimum	1,05	1,59	1,00	1,55
	Maximum	3,99	5,02	3,90	4,89
	Std. Deviation	0,63	0,85	0,61	0,77
Total	Mean	2,36	3,19	2,29	3,07
	Minimum	0,78	1,10	0,82	1,04
	Maximum	4,83	7,06	4,84	6,16
	Std. Deviation	0,81	1,02	0,83	0,97

### Agreement and concordance between the two spirometers

In order to evaluate agreement and concordance between the two devices, we calculated the Pearson correlation and the ICC for all the aforementioned spirometric parameters, between the two spirometers (Table 2). As we can see both metrics (Pearson correlation and ICC), and for all spirometric parameters considered is quite high (greater than 0.9), and for certain key spirometric parameters (FEV_1_, FVC, FEV_1_/FVC and FEF_25–75%_) is greater than 0.94.

**Table 2:** Pearson correlation coefficients and intraclass correlation coefficients (ICC) between the spirometric values obtained with the two spirometers, for the entire dataset (200 patients)

	Pearson correlation	ICC
*FEV*_*1*_*(L)*	0.976	0.976
*FVC (L)*	0.963	0.962
*FEV*_*1*_*/FVC*	0.947	0.945
*FEF*_*25–75%*_	0.953	0.948
*PEF (L/sec)*	0.922	0.922
*MEF*_*25%*_	0.909	0.906
*MEF*_*50%*_	0.944	0.942
*MEF*_*75%*_	0.946	0.942

*for all metrics p < 0.001

**Fig. 2:**
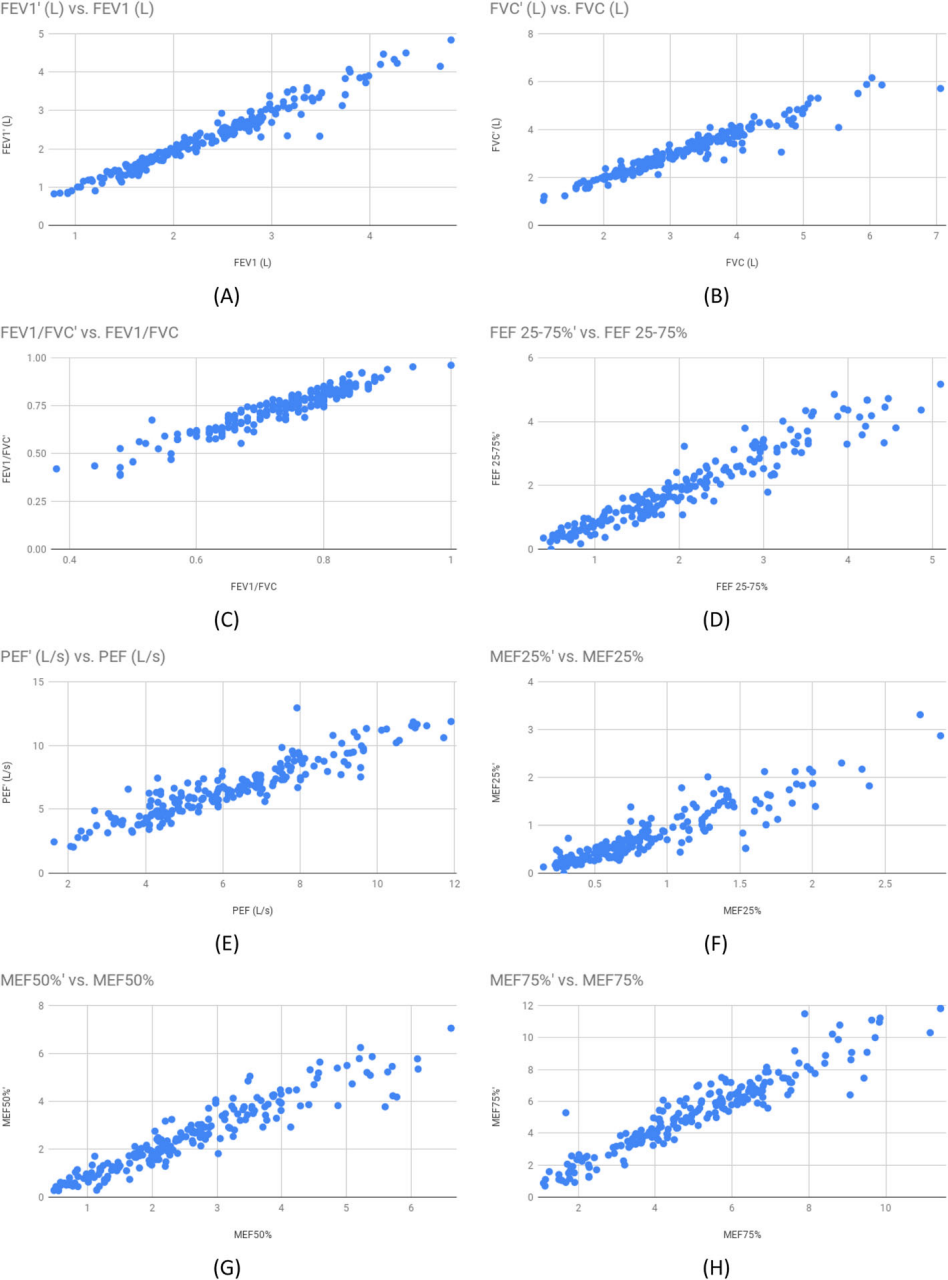
Correlation plots between the values obtained from the two spirometers, for the spirometric parameters considered in this work: (A) FEV_1_, (B) FVC, (C) FEV_1_/FVC, (D) FEF_25–75%_, (E) PEF, (F) MEF_25%_, (G) MEF_50%_, (H) MEF_75%_

The correlation plots for these parameters presented in Fig. 2 visually depict the high concordance between the two spirometers, on all calculated spirometric parameters. As exhibited in the plots there is significant agreement between the two spirometers, for all spirometric parameters, especially for FEV_1_, FVC, FEV_1_/FVC ratio and FEF_25–75%_ that are primarily useful for the interpretation of spirometry.

In the plots that follow FEV_1_, FVC, FEV_1_/FVC, FEF_25–75%_, PEF, MEF_25%_, MEF_50%_ and MEF_75%_ refer to the values obtained from the Air Next spirometer, whereas FEV_1_’, FVC’, FEV_1_’/FVC’, FEF_25–75%_’, PEF’, MEF_25%_’, MEF_50%_’ and MEF_75%_’ are the values obtained from the conventional spirometer.

In an additional exploratory analysis, we calculated Pearson’s correlation coefficients and ICC for each of the four patient subgroups, namely asthma, COPD, restrictive and normal, in order to gain further insight regarding the performance and validity of the measurements obtained with Air Next spirometer in each case. The respective results are shown in Table 3.

**Table 3:** Pearson correlation and ICC between the spirometric values obtained with the two spirometers, for each of the four patient subsets, namely: asthma, COPD, restrictive and normal

		Pearson correlation	ICC
**Asthma**	*FEV*_*1*_*(L)*	0.979	0.979
	*FVC (L)*	0.972	0.969
	*FEV*_*1*_*/FVC*	0.906	0.898
	*FEF*_*25–75%*_	0.943	0.943
	*PEF (L/sec)*	0.965	0.963
	*MEF*_*25%*_	0.836	0.835
	*MEF*_*50%*_	0.930	0.929
	*MEF*_*75%*_	0.963	0.959
**COPD**	*FEV*_*1*_*(L)*	0.968	0.967
	*FVC (L)*	0.924	0.914
	*FEV*_*1*_*/FVC*	0.914	0.916
	*FEF*_*25–75%*_	0.960	0.96
	*PEF (L/sec)*	0.926	0.925
	*MEF*_*25%*_	0.915	0.89
	*MEF*_*50%*_	0.961	0.959
	*MEF*_*75%*_	0.950	0.949
**Restrictive**	*FEV*_*1*_*(L)*	0.946	0.946
	*FVC (L)*	0.947	0.942
	*FEV*_*1*_*/FVC*	0.943	0.937
	*FEF*_*25–75%*_	0.903	0.895
	*PEF (L/sec)*	0.850	0.85
	*MEF*_*25%*_	0.730	0.725
	*MEF*_*50%*_	0.912	0.905
	*MEF*_*75%*_	0.887	0.884
**Normal**	*FEV*_*1*_*(L)*	0.971	0.968
	*FVC (L)*	0.975	0.974
	*FEV*_*1*_*/FVC*	0.900	0.899
	*FEF*_*25–75%*_	0.902	0.898
	*PEF (L/sec)*	0.914	0.914
	*MEF*_*25%*_	0.914	0.908
	*MEF*_*50%*_	0.857	0.856
	*MEF*_*75%*_	0.918	0.917

*for all metrics p < 0.001

It is evident both Pearson correlation and ICC are quite high for all spirometric parameters and in all patient subsets. Especially, for the most important parameters for interpreting a spirometry, i.e. FEV_1_, FVC, FEV_1_/FVC and FEF_25–75%_, nearly all values are greater than 0.9. It should be noted that for all calculated correlations the corresponding *p*-values were < 0.001.

**Fig. 3:**
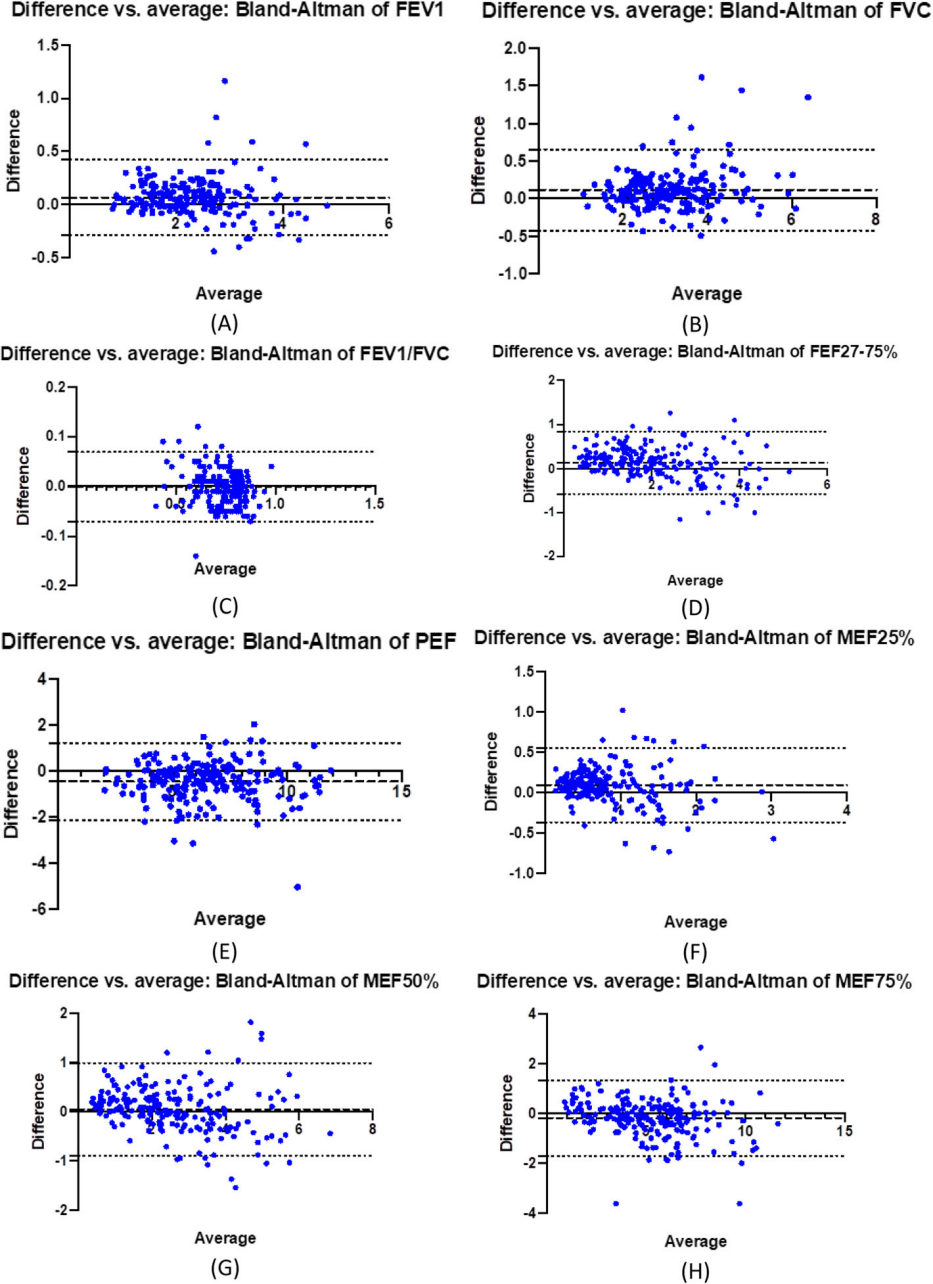
Bland-Altman plots for the evaluated spirometric parameters: (A) FEV_1_, (B) FVC, (C) FEV_1_/FVC, (D) FEF_25–75%_, (E) PEF, (F) MEF_25%_, (G) MEF_50%_, (H) MEF_75%_. Dashed lines represent the mean difference between measurements and dotted lines the 95% limits of agreement

In order to further evaluate the reproducibility of the measurements with the Air Next vs. the conventional spirometer, we have developed Bland-Altman plots (Fig. 3). In these plots, we provide a visualization of the mean difference of the evaluated spirometric parameters between the two spirometers. In all cases we observed a small mean difference between the two devices, with the majority of measurements being well within the limits of agreement. These plots support a good agreement between the two devices.

## Discussion

In this cross-sectional prospective study, we have shown that spirometric measurements with the ultra-portable Air Next spirometer present very good agreement (as expressed by Pearson’s and intraclass correlation coefficients > 0.94 for all evaluated parameters) and reproducibility (in Bland-Altman plots) with a standard desktop spirometer. Our results performed in the context of an outpatient clinic of a tertiary hospital in a wide range of patients with different spirometric patterns and healthy controls support the reliability of this novel ultra-portable spirometer.

Spirometry plays an integral role in the diagnosis and monitoring, primarily for respiratory diseases and conditions. Purchasing and maintaining an office based spirometer is costly and cumbersome; in addition, operating such a device requires a dedicated computer and trained personnel able to perform the spirometry and subsequently interpret the reported results. For these reasons, spirometers are largely underused in primary care [13]. The cost of spirometry programs is significant and referral to tertiary centers or specialty care is not always feasible and bears an additional significant cost [14]. Portable spirometers do not suffer from the aforementioned issues and offer an appealing low-cost solution for widespread adoption of spirometry, not only in primary care but in patients’ home as well.

The most crucial concern regarding the utilization of portable spirometers, is the quality of their measurements. Independent validation of medical devices is quite important in order to avoid any bias and ascertain reproducibility. To this end, in the current work we compared the spirometric parameters obtained by a conventional spirometer and the portable Air Next spirometer. In order to compare the two spirometers more systematically, we have compared all parameters included in their respective reports, even the ones that are not routinely used in the interpretation of spirometry. As for the patient set our aim was to achieve equal representation of major respiratory diseases, as well as healthy subjects, in order to avoid any bias and ensure reproducibility of the obtained results.

Based on the metrics and graphs shown previously, there is great concordance between the two spirometers. As for Pearson correlation and ICC we have also reported these metrics for each of the patient subsets, aiming to detect any perturbation within each category; however, the obtained results show that for all patient subsets, the two spirometers exhibited significant correlation, indifferent to the underlying condition or disease.

Similar studies have been previously presented in the literature for the validation of the portable Air Smart spirometer [15, 16], i.e. the predecessor of Air Next. Unlike Air Next that connects to a smart device wirelessly, the Air Smart spirometer featured a wired connection via a jack cable.

In the current work, all spirometries were performed by a trained nurse; since, the Air Next spirometer is also accessible and is intended for use by patients without technical or medical training, it would be rather interesting to see a validation were spirometries with the portable spirometer are performed by the patients themselves. In a similar sense, a future validation could be performed in the emergency department setting, or include primary care doctors and/or pharmacists.

## Conclusions

Portable spirometers feature a multitude of characteristics that makes them an ideal solution for extensive adoption in several medical and non-medical settings. Specifically, the Air Next spirometer is an ultra-portable, low cost spirometric device that does not need calibration and can be operated via a user-friendly smartphone application. Besides these practical characteristics, the most important feature of Air Next spirometer is the quality of reported results. After the careful and extensive validation performed in the current work, the results yielded by the Air Next and a conventional spirometer exhibit very good agreement and reproducibility. Our results support the use of Air Next as a reliable spirometer for the screening and diagnosis of various spirometric patterns in clinical practice.

## Data Availability

The datasets used and/or analysed during the current study are available from the corresponding author on reasonable request.
